# Meatotomy1Delivered at the Sisters of Charity Hospital, January 20, 1898.

**Published:** 1898-10

**Authors:** P. J. Candee


### Clinical Lecture.

### MEATOTOMY.[1]

By BYRON H. DAGGETT, M. D., Buffalo, N. Y.

Reported by P. J. Candee, M. D.

THE outlet of the urinary canal is called the meatus. Meatus is a Latin word signifying a passageway, hence it is given to the balanic or that portion of the canal embraced by the glans. The external meatus is the name given to the immediate or membranous opening. The orifice is located normally in the apex of the glans, and varies in size from a pin-hole to a vertical slit one-third of an inch or more in length. Its variation from the normal gauge may be

1. Delivered at the Sisters of Charity Hospital, January 20, 1898

congenital or may be due to acquired causes. If the lumen be sufficiently contracted to obscure free urination or necessary instrumentation, an operative procedure is in order. This operation usually consists in slitting through the lower angle of the meatus, and is called meatotomy.

The lumen of the balanic urethra is closed by coaptation of its lateral walls; in fact it has no lumen or caliber, except when in use. It gaps by pressure upon the glans and dilates with the intrusion or extrusion of foreign substances. This portion of the urinary canal is inelastic and possesses no surrounding or constricting muscular fibers. It becomes narrow or stenosed by thickening, infiltration and induration of its walls, hence incision and not dilatation or divulsion is employed for its enlargement. This section is the most sensitive and the narrowest portion of the normal canal, and is anatomically a complex structure. Here we find the fossa navicularis, numerous papillæ, mucous cysts, the valve of Guerin and folds of membrane, forming valves[1] upon the lateral walls of this passageway and constituting it a perfect nozzle for clean and free ejections of the genito-urinary secretions.

Harrison says that narrowing of this orifice often retards the healing of urethritis by confining the discharges and obstructing normal drainage. Otis confirms this opinion and has made a study of the normal caliber of the urethral canal. Otis also says that the strictured meatus should be cut in the lower angle or floor, for the reason that there is less liability of hemorrhage and less chance for recontraction and that there is more certainty of cutting the constringing bands. There is probably less liability of hemorrhage from the inferior cut, but the other objection is less tenable, since it is infiltration and thickening rather than constringing bands that cause the narrowing.

Meatotomy became the prevailing medical fad a few years ago, and many artificial hypospadias were made by amateur surgeons and aggressive doctors. There certainly is a choice in the locality of the incision, for if the floor of the canal is thinned, or already has a squint toward hypospadias, the cut should be made above and in some cases both above and below.

Stewart (Diseases of the Urethral Canal) says that the meatus should not be recklessly slit down through the frenum, as is often done.

Overcutting through this structure destroys nature's receptacle for retaining the normal secretion which supplies moisture and lubri-

---

1. See illustrated article printed in the New York *Medical Record*, May 7, 1898.

cation to the parts ; it weakens the force and broadens the urinary stream and causes dripping of the urine. Such an opening down through the meatal fourchette drains the natural secretion, just as a dependent opening draws off the saliva from the buccal cavity. Again, the diphtheritic membrane sometimes forms upon these raw and gaping surfaces. Such an operation not only impairs the function of this structure as a nozzle, but it also vitiates the appearance and presentability of the organ.

The external meatus is formed by the muco-cutaneous membrane ; it is slightly hooded at the upper angle and forms more or less of a fourchette at the lower angle. The urethra dips down to a greater or less degree behind the fourchette. The external meatus is quite elastic ; the balanic urethra is firm and inelastic, hence incision is the operation of choice for the enlargement of the balanic urethra, but the incision need not often involve the membranous outlet, which if incised at all it should be only nicked and cut less deeply than the rigid balanic urethra. This external meatus forms a gateway which protects the urethra from without as well as from within. Do not destroy its usefulness—it will serve and not offend if properly treated.

The appearance of the meatus is indicative of disturbance in the urethral way just as the tongue and throat often reflect disorders of the digestive tract. The bright red and swollen meatus indicates acute inflammation ; a dull red and patulous meatus indicates passive congestion ; a dark red and herpetic meatus with an elongated prepuce indicates prostatic hypertrophy ; a pinched appearance indicates some wasting disease of the genito-urinary tract.

Filaretopoulo says that examination of the urine for tubercle bacilli is frequently negative, and that infiltration and eversion of the lips, bluish tinge of the membrane, resistance to compression, a discharge aggravated by injections of nitrate of silver, urine at the same time being highly acid, are presumptive evidences of tubercular disease.

There is another curious fact which I have neglected to mention, and that is that many cases of contracture in the posterior urethra disappears like magic after the enlargement of the meatus.

---

Acute Follicular Tonsillitis.—Dr. E. Fletcher Ingals, of Chicago, recommends the application to the inflamed tonsil of a 50 per cent. solution of guaiacol in oil of sweet almonds. Internally :

℞ Potass. bromid. . . . . . . . . . . . . . . . . . . . . . . . . . . . . . . .gr. 80.

Sod. salicyl. . . . . . . . . . . . . . . . . . . . . . . . . . . . . . ʒ i.

Tr. opii deodor. . . . . . . . . . . . . . . . . . . . . . . . . . . . . ʒ i.

Cascara cordial ad. . . . . . . . . . . . . . . . . . . . . . . . . . ℥ i.

M. Sig. Teaspoonful every four hours in water.—*Chicago Clin. Rev.*

